# Healing Potential of the Marine Polysaccharides Carrageenan and Ulvan on Second-Degree Burns

**DOI:** 10.3390/jfb15090257

**Published:** 2024-09-05

**Authors:** Dimitra Statha, Asimina Papaioannou, Stefanos Kikionis, Maria Kostaki, Ioannis Sfiniadakis, Andreas Vitsos, Jane Anastassopoulou, Efstathia Ioannou, Vassilios Roussis, Michail Christou Rallis

**Affiliations:** 1 Section of Pharmaceutical Technology, Department of Pharmacy, National and Kapodistrian University of Athens, Panepistimiopolis Zografou, 15784 Athens, Greece; demetrastatha@gmail.com (D.S.); asimina29@gmail.com (A.P.); marie.kstk@gmail.com (M.K.); avitsos@pharm.uoa.gr (A.V.); i.anastassopoulou@gmail.com (J.A.); 2 Section of Pharmacognosy and Chemistry of Natural Products, Department of Pharmacy, National and Kapodistrian University of Athens, Panepistimiopolis Zografou, 15771 Athens, Greece; skikionis@pharm.uoa.gr (S.K.); eioannou@pharm.uoa.gr (E.I.); roussis@pharm.uoa.gr (V.R.); 3 Pathologoanatomic Laboratory, Athens Naval Hospital, 11521 Athens, Greece; jsfiniadakis@yahoo.gr

**Keywords:** partial-thickness burn, *in vivo* mice model, carrageenan, ulvan, wound care, infrared spectroscopy

## Abstract

The treatment of second-degree burn wounds presents a significant clinical challenge, often characterized by prolonged healing times and risk of complications. In this study, the wound healing potential of bioactive marine sulfated polysaccharides ulvan and carrageenan formulated in gels at concentrations of 1.5%, 5.0%, and 10% w/w was evaluated. Hairless female SKH-hr2 mice (n = 7 per treatment) with burn-inflamed skin were treated with the polysaccharide-based gels, and the therapeutic efficacy was assessed using a comprehensive array of evaluation methods, including a histopathological analysis, clinical observation, photo-documentation, an image analysis, an evaluation of biophysical skin parameters, and FT-IR spectroscopy. Our findings indicate that the 10% w/w carrageenan gel exhibited significant enhancement in wound healing, particularly in the early stages of the healing process. This was evidenced by the restoration of the α-helix structure of collagen and the configuration of glycosaminoglycans, as demonstrated by FT-IR absorption bands of the skin both *in vivo* and *ex vivo*. Furthermore, the 5% w/w ulvan gel also demonstrated notable efficacy in promoting wound healing, particularly in the later stages of the healing process. These results suggest that carrageenan and ulvan gels hold promise for improving the efficiency of wound healing in second-degree burn wounds. Our study contributes to the understanding of the therapeutic potential of marine polysaccharides and provides insights into their mechanism of action in promoting wound healing.

## 1. Introduction

Burn wounds represent a major public health issue with significant morbidity and mortality. They are associated with prolonged hospitalization, disfigurement, disability, and social stigma, while in severe cases their outcome can be fatal, as according to the World Health Organization (WHO), approximately 11 million burn injuries and 180,000 deaths related to severe burns are estimated to occur annually worldwide [1,2,3,4]. They are among the most common injuries of the skin and depending on the depth of skin injury, they are classified as superficial/epidermal (first degree), partial-thickness (second degree), and full-thickness (third degree) burns [5]. Among these, second-degree burns are particularly common in clinical practice and pose significant challenges in management with a tendency to result in severe infections, especially when they involve large areas of the body or when timely treatment is not provided. Managing second-degree burns often requires a multifaceted approach. Antibiotics may be prescribed, particularly in cases of infection or when individuals are deemed at a high risk of developing one, while severe infections may necessitate intravenous antibiotic therapy. Additionally, very severe cases may require skin grafting to facilitate optimal wound healing. Despite the prevalence and clinical significance of second-degree burns, there is currently no standardized therapeutic protocol for their management. This underscores the pressing need for further research and the development of evidence-based guidelines to improve outcomes for individuals affected by these injuries [2,3,4,5].

Wound healing is a physiological process that occurs as a natural response of the body to restore its injured tissues. It is a dynamic and complex process consisting of four interconnected and often overlapping phases that include hemostasis, inflammation, proliferation, and remodeling. Characterized by various biophysiological functions and different predominant cells, the proper sequence, duration, and intensity of these phases are crucial for the complete healing of wounds [6]. With several biological events and biochemical components involved in each phase, the breakdown of this well-coordinated sequence results in incomplete wound healing, affecting ultimately the functional recovery of the wound [6].

In order to promote the healing process, a variety of wound treatments, primarily dressings, have been developed over the years. Wound dressings should have the ability to restore the skin barrier function, prevent infection, manage fluid loss, control the excess of exudates, and reduce scar formation [7]. The selection of suitable materials that can promote wound healing is of high importance for designing effective wound dressings.

Recently, marine polysaccharides have emerged as valuable biomaterials for the development of advanced wound dressing systems [8,9,10,11]. Marine polysaccharides are highly biocompatible, biodegradable, and abundant biopolymers with diverse structures and functionalities [12,13]. Exhibiting unique physicochemical properties and a broad spectrum of biological activities, they are attracting considerable attention for their utilization as safe biomaterials in various novel biomedical systems [14,15,16,17,18]. Due to their ability to absorb wound fluids and maintain a moist environment in the wound area, they are considered ideal wound dressing components promoting various phases of endogenous wound healing in different types of wounds when formulated in films, fibers, and hydrogels [9,19].

Among them, ulvans, amply present in the cell walls of green macroalgae of the order Ulvales, are complex anionic sulfated polysaccharides that are mainly composed of sulfated rhamnose, glucuronic and iduronic acids, and xylose [20,21,22]. Exhibiting a chemical analogy to glycosaminoglycans, intrinsic cytocompatibility, and significant antioxidant, anti-inflammatory, antiviral, antihyperlipidemic, and antitumor activity, ulvans are attractive as biomaterials for the development of hybrid composites, particles, nanofibrous matrices, hydrogels, multilayer films, membranes, and polyelectrolyte complexes [21,23,24,25,26,27]. Due to the presence of rare sugars, such as sulfated rhamnose and iduronic acid that may enhance their wound healing potential, they are considered a promising biopolymer for the development of novel wound dressing systems for the treatment of various skin inflammations [11,28,29].

Carrageenans are the main components of the cell walls of many red seaweeds. They are anionic sulfated polysaccharides composed of alternating units of D-galactose and 3,6-anhydro-D-galactose linked by α-(1,3) and β-(1,4) glycosidic bonds and depending on the sulfation degree and the 3,6-anhydro-D-galactose content, they are classified into three principal forms, namely kappa (κ)-, iota (ι)-, and lambda (λ)-carrageenans [10,30,31,32,33,34]. Due to their low cost, resemblance to native glycosaminoglycans, and significant biological activities (antioxidant, antihyperlipidemic, anticoagulant, antiviral, antitumor, and immunomodulatory), they are widely used in the food, cosmetics, and pharmaceutical sectors [30,31,35,36]. Among them, κ-carrageenans exhibiting enhanced gelling properties are considered a promising biopolymer for tissue regeneration and wound healing applications [10,35,37,38].

Carrageenans at concentrations ranging from 0.5 to 1.5% w/w have shown potential wound healing enhancement both *in vivo* [39,40] and *in vitro* [41]. However, carrageenans have never been tested at higher doses. Additionally, ulvans have shown *in vitro* antioxidant and antibacterial properties at concentrations of 5%, 7.5%, and 10% w/w [27].

The lack of effective healing agents for second-degree burns highlights the urgent necessity for further investigation and the establishment of evidence-based protocols to enhance outcomes for those afflicted by such injuries. As a preliminary step in this direction, our study aimed to explore the healing efficacy of ulvan and carrageenan, formulated into gels for topical application, in the treatment of second-degree burn wounds in mice. Gels of 4% polyacrylamide, C13-14 isoparaffin, and laureth-7 (PIL), incorporating ulvan or carrageenan at 1.5, 5, and 10% w/w, to define the optimal beneficial dose, were prepared and evaluated for their wound healing efficacy on the burn-inflamed skin of SKH-hr2 hairless female mice. The exploration into gel formulations serves as a preliminary evaluation of these materials, which could potentially be formulated into dressings in future studies.

The aim of the current study was to evaluate and compare the therapeutic effect of the two polysaccharides and possibly determine the optimum dose. To address that, skin histopathological analyses and clinical evaluation were performed. In addition, wound area, skin texture, and hemoglobin levels were determined using an image analysis, while biophysical skin parameters, such as transepidermal water loss (TEWL), hydration, and skin thickness, were also assessed. Moreover, the wound healing efficacy of the biopolymer-based gels was evaluated by recording *in vivo* the changes in the skin of mice before, during, and at the end of the treatment, by Fourier transform infrared (FT-IR) spectroscopy.

## 2. Materials and Methods

### 2.1. Isolation and Characterization of Marine Polysaccharides

Specimens of the green alga *Ulva rigida* were collected from the island of Kefalonia, Greece (GPS coordinates 38°20′ N, 20°44′ E). After cleaning thoroughly from foreign materials, the algal specimens were washed under seawater and fresh water, air-dried, and finally milled in small pieces. For the extraction of ulvan,100 g of the milled algal biomass was soaked in 2 L of distilled H_2_O and heated in an autoclave for 20 min at 121 °C. Subsequently, the hot aqueous extract was filtered through a cotton cloth, and the resulting filtrate was left to cool at room temperature. For the precipitation of the polysaccharide, ethanol (96% v/v) (Sigma-Aldrich, St. Louis, MO, USA) was added to the filtrate (four times the volume of the filtrate) and the suspension was stored overnight at 4 °C. Finally, the resulting precipitate was filtered through a cotton cloth, washed with ethanol, sonicated for 60 min in an ultrasonic bath (AU-65 Analogic ultrasonic cleaner, Argo Lab, Carpi, MO, Italy), filtered under vacuum, and lyophilized overnight to afford ulvan in the form of an off-white powder, which was milled before use. The characterization of ulvan was performed as previously described [28]. The isolated ulvan had a molecular weight distribution centered at approx. 1080 kDa and a sulfate content of 48.2% on a dry weight basis. The FT-IR spectrum of ulvan exhibited characteristic absorption bands at 3350, 1610, and 1030 cm^−1^ attributed to the stretching vibrations of -OH, -C=O, and C-O-C groups, respectively, while the characteristic absorption bands of the S=O and C-O-S groups were observed at 1208 and 845 cm^−1^, respectively.

Specimens of the red alga *Hypnea musciformis* were collected from the island of Ithaca, Greece (GPS coordinates 38°26′ N, 20°41′ E). After cleaning thoroughly from foreign materials, the algal specimens were washed under seawater and fresh water, air-dried, ground into powder, and finally subjected to Soxhlet extraction with methanol (Sigma-Aldrich, St. Louis, MO, USA) for the removal of pigments. For the extraction of carrageenan, 100 g of the depigmented, dried algal biomass was soaked in 15 L NaOH (1 M) (Sigma-Aldrich, St. Louis, MO, USA) and heated for 3 h at 90 °C under stirring. After the adjustment of the pH to 8 by the addition of HCl (Sigma-Aldrich, St. Louis, MO, USA), the hot aqueous extract was filtered through a cotton cloth, and the filtrate was concentrated under reduced pressure to half of its volume. For the precipitation of the polysaccharide, 96% ethanol (v/v) was added to the filtrate (twice the volume of the concentrated filtrate) and the suspension was stored overnight at 4 °C. Following centrifugation (5000 rpm, 20 min) of the suspension at room temperature, the precipitate was washed with ethanol, sonicated for 60 min in an ultrasonic bath, filtered under vacuum, and lyophilized overnight. Subsequently, the obtained crude polysaccharide after being dissolved in distilled H_2_O was dialyzed using a cellulose membrane for 24 h against dd. H_2_O, and finally lyophilized overnight to afford pure carrageenan in the form of off-white flakes. The isolated carrageenan, which was identified as κ-carrageenan, had a molecular weight distribution centered at approx. 290 kDa, and a sulfate content of 29.6% on a dry weight basis. The FT-IR spectrum of κ-carrageenan displayed characteristic absorption bands at 3369, 1224, and 1036 cm^−1^ attributed to the stretching vibrations of -OH, -S=O, and C-O-C groups, respectively. The absorption bands at 928 cm^−1^ and 845 cm^−1^ indicated the presence of 3,6-anhydro-D-galactose and D-galactose-4-sulphate, respectively [37,42,43,44].

### 2.2. Preparation of Topical Gels

PIL (polyacrylamide, C13-14 isoparaffin, and laureth-7) (Seppic, Courbevoie, France) was used for the preparation of the gels, as an increasing viscosity factor. The topical gels were prepared by dissolving ulvan or carrageenan at concentrations of 1.5%, 5%, and 10% w/w in water for injection (Demo, Krioneri, Greece) followed by a gradual addition of PIL (4% w/w). Subsequently, the obtained formulations were homogenized using a VirTis TEMPEST VirTishear homogenizer (Gardiner, Urbandale, IA, USA) and stored at 2–6 °C.

### 2.3. Animals

The experimental procedures were performed in accordance with the animal care guidelines established by the European Council Directive 2010/63/EU. The experiment was carried out after the positive opinion of the Animal Protocols Evaluation Committee and the approval from the Greek Peripheral Veterinary Authority (protocol number: 775329/09-08-22. Female SKH-hr2 hairless mice (2–5 months, 25–30 g) were obtained from the Small Animal Laboratory of the Section of Pharmaceutical Technology, Department of Pharmacy, National and Kapodistrian University of Athens. The animals were housed under laboratory conditions in a controlled environment (temperature of 22–25 °C and humidity of 30–55%) with a 12 h light–12 h dark cycle with food (Nuevo S.A., Schimatari, Greece) and water available ad libitum. Prior to the experiment, the mice were acclimatized for 10 days.

### 2.4. Burn Wound Model and Study Design

Mice were anesthetized by the intraperitoneal administration of 100 mg/kg ketamine (Narketan 10, 100 mg/mL, Vetoquinol SA, Lure Cedex, France) and 7 mg/kg xylazine (Xylapan, 20 mg/mL, Vetoquinol SA, Lure Cedex, France). Burn wounds were induced on the upper dorsal skin of mice, 2 cm below the ears, using a circular metal stamp of a 2 cm^2^ area, priorly immersed in a water bath of 69 ± 2 °C for 2 s and immediately pressed on the skin for 10 s. This procedure, according to prior histopathological assessment of the burn wound severity, induces second-degree burns [45].

All animals received paracetamol (Vianex, Athens, Greece) dissolved in their water (1.0 mg/mL) for 3 days to relieve pain. The gels were applied once per day and the burn wound was covered by Fixomull Stretch Self Adhesive Gauze (BSN Medical, Hamburg, Germany) combined with Medicomp non-woven gauze (Hartmann, Hamburg, Germany). The adhesive was cut in a hexagon shape to prevent restricting the movement of the mice. The experiment was terminated when complete wound closure was achieved for ≥80% of mice in one of the treatments. For the wound healing evaluation, the study design included eight groups, each consisting of seven mice (n = 7). One group was the control group that did not receive any gel application; the second was the vehicle group that received the gel without any active ingredients; the third, fourth, and fifth groups were treated with the gel containing ulvan at 1.5%, 5%, and 10% w/w, respectively; and the sixth, seventh, and eighth groups were treated with the gel containing carrageenan at 1.5%, 5%, and 10% w/w, respectively (Table 1).

### 2.5. Burn Wound Healing Assessment

#### 2.5.1. Clinical Evaluation and Assessment of Wound Area, Skin Texture, and Hemoglobin

The mice were subjected daily to macroscopic examination, and photo-documentation was conducted using a Nikon D5100 digital camera equipped with an AF-S Micro Nikkor 60 mm f/2.8 G ED lens (Nikon, Tokyo, Japan) on the first and every two days. The digital camera was positioned at a fixed distance of 20 cm perpendicular to the subject.

An Antera 3D camera (Miravex, Dublin, Ireland) was used at the beginning, every other day, and the end of the experiment to measure the wound area (mm^2^) and evaluate skin texture and hemoglobin levels. The Antera 3D image analysis system used light-emitting diodes (LEDs) in order to achieve a high-precision analysis [46]. The healing rate was expressed using linear regression to analyze the reduction in the mean wound area for each group. This analysis was conducted separately for two distinct time periods: from day 1 to day 15, and from day 15 to day 26.

#### 2.5.2. Assessment of Biophysical Skin Parameters

Non-invasive biophysical methods were used to evaluate transepidermal water loss (TEWL), hydration, and skin thickness, before the burn induction and after treatment. TEWL (g/m^2^/h) and hydration (AU) were measured using Tewameter TM 300 (Courage + Khazaka Electronic GmbH, Cologne, Germany) and Corneometer CM820 (Courage + Khazaka Electronic GmbH, Germany), respectively. Skin thickness (mm) was measured using an electronic digital caliper (Powerfix Prof Milomex Ltd., Cardiff, UK). Before every measurement, the skin was thoroughly cleaned by wiping with sterilized gauze.

#### 2.5.3. Histopathological Analysis

At the end of the experiment, the mice were sacrificed by cervical dislocation and skin samples of the burn wound area were obtained and evaluated histopathologically. The skin samples were immersed in a 37% formaldehyde solution, embedded in paraffin wax, and sectioned at a 5 µm thickness. The multi-head light microscope NIKON eclipse 50 (Nikon Corp., Tokyo, Japan) was used for examination. Sections were stained by hematoxylin–eosin and photographed under 100× magnification, to blindly evaluate the extent of inflammation, edema, hyperkeratosis, and wound thickness, as well as the presence of ulceration, necrosis, and parakeratosis. The evaluation was based on the criteria shown in Table 2.

#### 2.5.4. FT-IR Spectra

FT-IR spectroscopy is considered a valuable, sensitive, low-cost, fast, and non-invasive technique for the characterization and identification of the molecular structures of human skin tissues and other complex systems. FT-IR can provide information for all components of biological tissues or liquid samples. The advantage of FT-IR spectroscopy is that all components of the samples are recorded simultaneously. Additionally, biological samples do not require any special preparation, such as coloring or demineralization, as needed in classical histopathology [47]. The FT-IR spectra were recorded *in vivo* by using the 4300 handheld FT-IR spectrometer (Agilent Technologies, Petaling Jaya, Malaysia) equipped with an attenuated total reflection (ATR) crystal, with a resolution of 4 cm^−1^ and 60 scans/spectrum.

#### 2.5.5. Statistical Analysis

All data were tested concerning normality using the Shapiro–Wilk test. The statistical significance between the groups was determined by Student’s *t*-test, one-way analysis of variance (ANOVA), and their non-parametric equivalents Wilcoxon-rank test and Kruskal–Wallis test, respectively. A *p*-value of <0.05 was considered statistically significant. The statistical analysis and graph creation were conducted using GraphPad Prism 8.4.3 (GraphPad Software, Inc., San Diego, CA, USA).

## 3. Results

### 3.1. Clinical Evaluation

**Figure 1:**
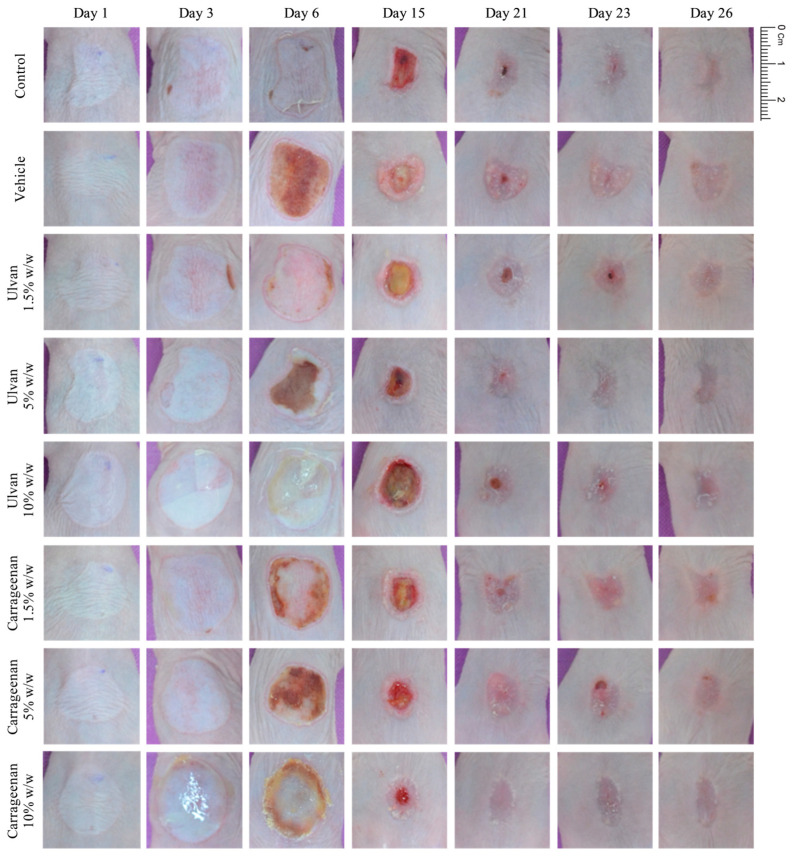
Representative images depicting the wound areas of mice receiving no treatment; vehicle; 1,5%, 5%, and 10% w/w of ulvan gels; and 1,5%; 5%, and 10% w/w of carrageenan gels, captured on days 1, 3, 6, 15, 21, 23, and 26. Among the treatment groups, mice treated with the 10% w/w carrageenan gel exhibited the most favorable clinical outcomes followed by those treated with 5% w/w ulvan gel.

Representative digital images of burn wound areas of mice receiving different medications, at various concentrations, are presented for days 1, 3, 6, 15, 21, 23, and 26 of treatment in Figure 1. A comparison among these images revealed that mice treated with the 10% w/w carrageenan gel and the 5% w/w ulvan gel exhibited the highest healing efficacy.

From these images, it was observed that on day 21, the 10% w/w carrageenan gel showed full wound closure in most of the mice, while 1.5 and 5% w/w carrageenan gels showed, comparatively, no particular healing effect.

Ulvan gel at the concentration of 5% w/w also exhibited significant healing efficacy. On day 23, the 5% w/w ulvan gel showed full wound closure in most of the mice, while ulvan gels at concentrations of 1.5 and 10% w/w showed no healing effect.

The vehicle, in relation to the control, appeared to be slightly toxic, showing intense erythema and edema at the period of days 8–13 and hypertrophic scar formation.

### 3.2. Assessment of the Burn Wound in Relation to the Recovery of the Damaged Area

**Figure 2:**
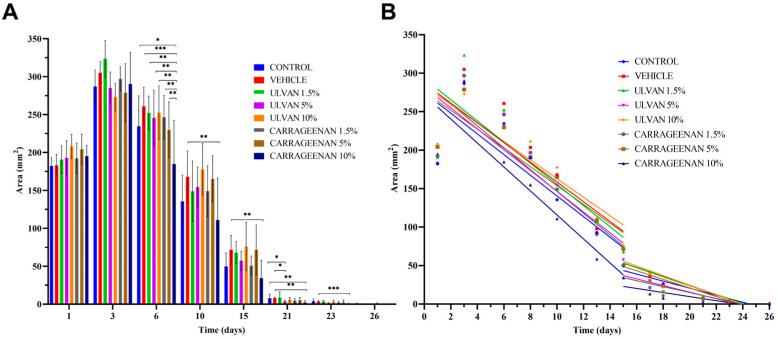
(**A**) Histogram of wound area reduction in relation to time. (**B**) Wound healing rate expressed using linear regression analysis for two distinct time periods, i.e., days 1 to 15, and 15 to 26; * *p* < 0.05, ** *p* < 0.01, *** *p* < 0.001.

Figure 2A depicts the histograms of wound area reduction, while Figure 2B shows the rate of reduction in the damaged wound area resulting from the linear regression analysis (Table 3).

Figure 2A indicates that on day 6, the 10% w/w carrageenan gel significantly decreased the wound area in relation to the control and the other treatments (*p* < 0.05). On day 23, mice receiving the 10% w/w carrageenan gel still had significantly smaller wounds than those treated with the gel consisting only of the excipients.

Linear regression analysis data revealed that during the first half of the healing process (days 1–15), the 10% w/w carrageenan gel exhibited the highest healing rate (Table 3). Ulvan appeared to have a beneficial impact during the latter half of the healing process (days 15–26).

### 3.3. Histopathological Evaluation

**Figure 3:**
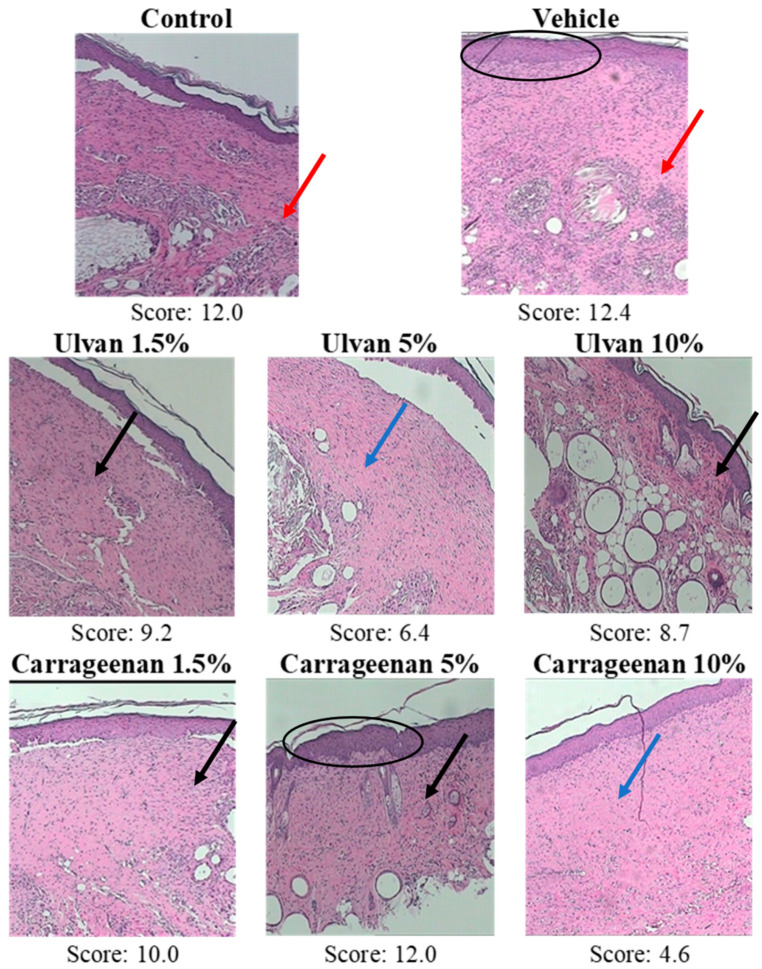
Hematoxylin and eosin-stained sections of mice skin at the burn wound area for mice receiving different treatments (100×). Blue arrows depict mild inflammation in the 10% w/w carrageenan and 5% w/w ulvan groups; black arrows depict moderate inflammation in the 1.5% and 10% w/w ulvan groups, as well as in the 1.5% and 5% w/w carrageenan groups; red arrows depict severe inflammation in the control and vehicle groups; ellipses depict epidermal hyperplasia in the vehicle and 5% w/w carrageenan group.

The results of skin biopsies concerning inflammation (Infl), edema (Oed), hyperkeratosis (HPKe), wound thickness (WTh), ulceration and necrosis (Ulce&Ne), and parakeratosis (PKe) are presented in Figure 3 and Table 4. Based on these data, it was concluded that treatment with the 10% w/w carrageenan (score 4.6) and the 5% w/w ulvan (score 6.4) gels reduced inflammation, and no significant hyperkeratosis of the overlying epidermis was observed.

On the contrary, treatments with the 1.5% w/w ulvan (score 9.2), 10% w/w ulvan (score 8.7), 1.5% w/w carrageenan (score 10.0), and 5% w/w carrageenan (score 12.0) gels gave slightly better histological evaluation than the control (score 12.0), showing moderate inflammation, edema, and hyperemic vessels. In the case of the vehicle (score 12.4), severe inflammation, with intense dilatation, and hyperemia of the vascular branches, as well as intense inflammatory infiltrations with the presence of lymphocytes, plasma cells, and numerous polymorphonuclear leukocytes, was detected. According to the scoring system described in Table 2, the 10% w/w carrageenan gel achieved the lowest score and the vehicle the highest (Figure 3).

### 3.4. Assessment of Hemoglobin Levels and Skin Texture

**Figure 4:**
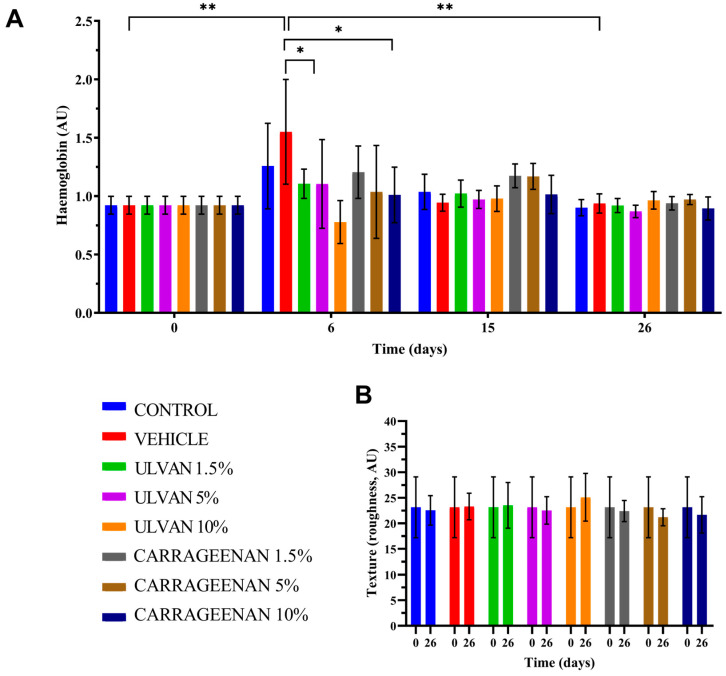
Histograms of (**A**) hemoglobin and (**B**) skin texture of mice receiving different treatments in relation to time (* *p* < 0.05, ** *p* < 0.01).

The histograms of hemoglobin and skin texture of mice, receiving different treatments in relation to time, are presented in Figure 4. The analysis of hemoglobin levels showed that on day 6, when the wounds attained their maximum size, the vehicle group had significantly higher hemoglobin values compared to the 5% w/w ulvan and 10% w/w carrageenan groups, which was associated with intense inflammation. The hemoglobin levels of the various treatments presented no statistically significant differences between the first and last day of the experiment (Figure 4A).

Concerning the skin texture, on the last day of the experiment, all treatments returned to their initial values (Figure 4B).

### 3.5. Evaluation of Biophysical Parameters

**Figure 5:**
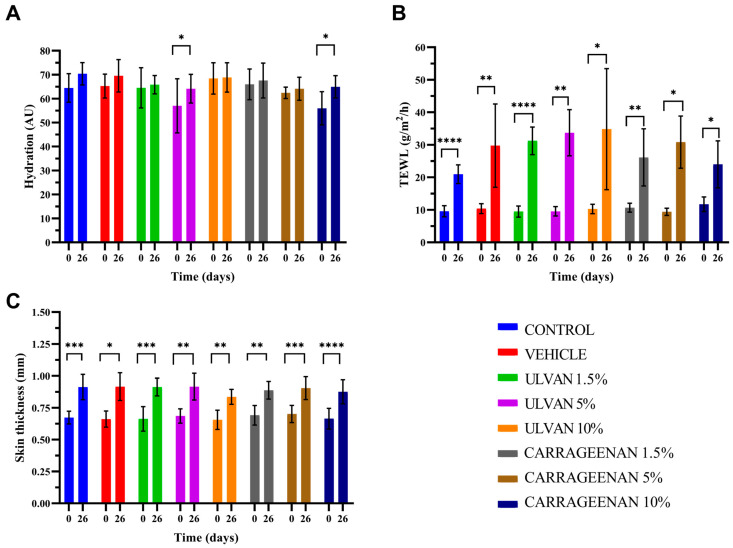
Histograms of (**A**) hydration, (**B**) transepidermal water loss (TEWL), and (**C**) skin thickness of different treatments at the beginning and the end of the experimental procedure (* *p* < 0.05, ** *p* < 0.01, *** *p* < 0.001, **** *p* < 0.0001).

The biophysical parameters of skin hydration, TEWL, and thickness in relation to time are presented in Figure 5.

The analysis showed that on day 26, the hydration of the mice skin treated with the 5% w/w ulvan and 10% w/w carrageenan gels was significantly higher compared to day 0, while in all other cases, the hydration returned to normal (Figure 5A). TEWL did not recover to normal with any of the treatments, which indicates that the skin barrier did not return to its normal structure (Figure 5B). Skin thickness was significantly increased in all cases, which is justified after the burn injury (Figure 5C).

### 3.6. FT-IR Spectroscopic Analysis of Mice Skin

**Figure 6:**
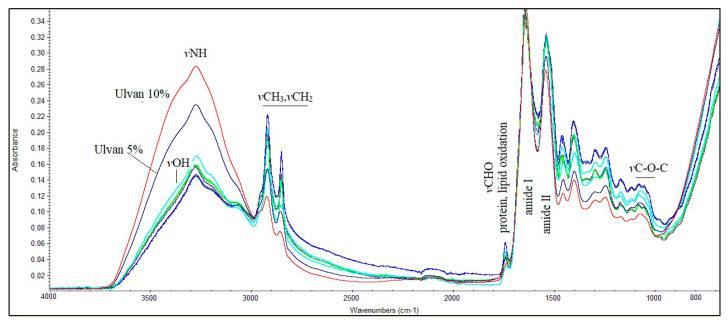
FT-IR spectra of mice skin treated with carrageenan and ulvan at various concentrations during the final stage of wound healing.

To identify the changes in skin tissue biological components at a molecular level after therapeutic application, FT-IR spectra were recorded in all mice, using a handheld FT-IR spectrometer, which provided the advantage to record, *in vivo*, the skin changes before, during, and at the end of the therapeutic schemes. Figure 6 presents representative FT-IR spectra of the skin before and at the end of treatment with carrageenan and ulvan at various concentrations.

The FT-IR spectra showed an important band intensity and shift differences across the entire spectral region between 4000 and 800 cm^−1^. It was also observed that the mice did not react to the wound healing treatment in the same way.

**Figure 7:**
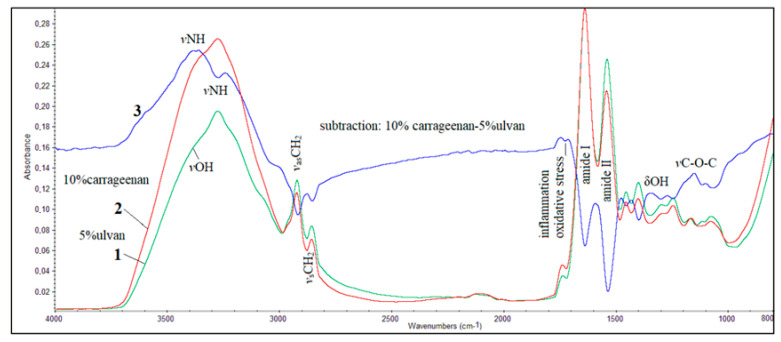
FT-IR spectra of mice skin during wound healing: (**1**) spectrum of mice skin treated with 5% w/w ulvan gel, (**2**) spectrum of mice skin treated with 10% w/w carrageenan gel, and (**3**) spectrum resulting from subtraction of spectrum **1** from spectrum **2**.

Since the clinical and histological results showed that the 10% w/w carrageenan and 5% w/w ulvan gels significantly enhanced the wound healing process, we compared the FT-IR spectra of the skin of mice treated with the corresponding gels (Figure 7).

The observed positive absorption bands in the subtracted spectrum indicated that, in skin treated with the 10% w/w carrageenan gel, the α-helix conformation of the protein secondary structure predominates. The shift of the *v*NH absorption bands of proteins in the spectrum of skin treated with the 5% w/w ulvan gel from about 3300 cm^−1^, typical for normal protein secondary structure, to a lower frequency at 3237 cm^−1^, combined with the increased intensity at 2922 cm^−1^ and 2858 cm^−1^ assigned to *vas*CH_2_ and *vs*CH_2_, respectively, indicated that after treatment, cell membranes became more lipophilic. This lipophilic environment promoted the production of aggregates, leading to protein misfolding and the adoption of a β-sheet secondary conformation by the proteins [47]. These observations were confirmed by the other measurements, where it was found that the skin was dehydrated. The negative peaks in the amide I and amide II absorption bands of the subtracted spectrum confirm that the skin of the mice treated with the 5% w/w ulvan gel exhibits the formation of protein β-sheets, attributed to the wound and inflammation of the skin. This was further confirmed by the “diagnostic band” at approximately 1740 cm^−1^. The most important indicator of the curative effect of the 10% w/w carrageenan gel was the appearance of the band in the region 1230–1000 cm^−1^, which is assigned to *v*C-O-C attributed to aminoglycans, indicating the formation of collagen and elastin.

## 4. Discussion

Clinical evaluation demonstrated that the mice treated with the 10% w/w carrageenan gel showed significantly enhanced wound healing in comparison to all other groups. The mice in this group approached almost complete wound closure, earlier than the other groups, from day 21. Mice treated with the 5% w/w ulvan gel also demonstrated a significant healing effect, ranking second to the group that was treated with the 10% w/w carrageenan gel, as they approached total wound healing on day 23.

During the experiment, the wound area was measured on various days and the healing rate was calculated. The measurement of wound areas revealed that mice treated with the 10% w/w carrageenan gel exhibited significantly smaller wounds compared to all other groups starting from day 6. This statistically significant difference in wound area was maintained for the vehicle group until the end of the experiment.

The 10% w/w carrageenan gel accelerated the healing process significantly in the first 15 days of the experiment, having the greatest slope compared to all other treatments. The 5% w/w ulvan gel appeared to accelerate the healing process during the second period, from day 15 to 26. It seems that the process of wound healing can be divided into two distinct phases, each progressing at a different rate. The initial phase is characterized by a faster rate (days 1–15), followed by the second phase, which exhibits a slightly slower progression (days 15–26).

Histopathological examination and hemoglobin measurements indicated that mice treated with the 10% w/w carrageenan gel and the 5% w/w ulvan gel exhibited reduced inflammation levels compared to the other groups. This observation highlights the significance of the dosage in relation to therapeutic effectiveness. The excipient, on the other hand, displayed more intense inflammation than all the other treatments, including the control. It is, therefore, not unlikely that the utilization of this gelling agent might have attenuated the therapeutic efficacy of the polysaccharides.

Concerning the biophysical parameters, the 10% w/w carrageenan and the 5% w/w ulvan treatments resulted in significantly higher hydration values, whereas TEWL and skin thickness were not restored to their initial levels in any of the groups.

The optimum efficacy of the 10% w/w carrageenan gel compared to the 5% w/w ulvan gel was also observed in the FT-IR spectra of the mice skin. As noted in the spectrum of the mice treated with the 10% w/w carrageenan gel, the increase in the intensity of the *v*NH and *v*OH absorption bands at approximately 3500 cm^−1^ and 3300 cm^−1^ indicated the formation of aminoglycans. Additionally, the band at 1772 cm^−1^, assigned to Ig-COO^-^, suggested the presence of inflammation. Negative peaks of amide I and amide II bands in the subtractive spectrum revealed that, although the histopathological data were similar, the proteins did not attain the natural α-helix secondary conformation after the application of the 5% w/w ulvan gel. A crucial observation was the increased intensity of the band in the spectral region between 1200 and 1000 cm^−1^, where the *v*C-O-C of sugar rings and bridges absorb, confirming the development of collagen and elastin.

Apparently, the dosage is closely related to the therapeutic effectiveness. Carrageenan exhibited optimal performance at the highest of the tested concentrations (10% w/w), while ulvan exhibited this at an intermediate concentration (5% w/w).

It has been suggested that carrageenan could be used in films as a wound healing agent [10], and its wound healing efficacy has been proven by various *in vitro* [41,48] and *in vivo* studies [39,40]. The beneficial effects of ulvan in burn wounds have been also reported in an *in vivo* study where the combination of ulvan with gelatin formulated in electrospun nanofibers showed a significant burn healing effect [45].

The mode of action of these sulfated polysaccharides on burn wounds is most probably multiparametric. Both carrageenan and ulvan possess antioxidant, anti-inflammatory, and immunomodulatory properties. Besides preventing or reducing inflammation, they absorb water to form hydrogels, continuously providing moisture over an extended period of time and decreasing the skin temperature, thereby reducing the extent of the injury, protecting the basal membranes, and reducing scarring [49]. Furthermore, through water release and the prevention of excessive oxidative stress, they support the viability of fibroblasts and keratinocytes, reducing necrosis [49].

Despite the uniform burns inflicted at the beginning of the experiment, mice treated with the vehicle exhibited a poorer clinical outcome compared to the control. With the vehicle, keloids and edema developed during the healing process, a fact that complicated the removal of necrotic tissue. Apparently, the gelling agent (PIL) is responsible for the observed secondary effects. Even though according to the literature, this gelling agent is non-toxic to normal skin, except for a single case of allergic contact dermatitis [50], it seems that the use of PIL on an open wound may cause this reaction. Therefore, in a future study, we plan to develop gels exploring the use of alternative gelling agents, optimizing the dose, and even combining the two polysaccharides to capitalize on the fact that carrageenan seems to be especially effective in the first half of the healing process, while ulvan is more effective in the second half. In parallel, even though the measurement of biophysical parameters is indicative, we will opt to incorporate the analysis of relevant biomarkers in an effort to better understand the mechanism of activity of these two polysaccharides.

## 5. Conclusions

Based on the results derived from the clinical observations, histopathological data, photo-documentation, and measurement of biophysical parameters, the 10% w/w carrageenan gel significantly improved burn wound healing, especially in the early stages. Following closely was the 5% w/w ulvan gel, which showed a significant effect, especially in the second half of the healing process. The gelling agent (polyacrylamide, C13-14 isoparaffin, and laureth-7) exhibited moderate topical toxicity in burns. The FT-IR spectra confirmed the elastin and collagen formation upon the application of the 10% w/w carrageenan gel on skin. The present study revealed that the 10% w/w carrageenan gel, and secondarily, the 5% w/w ulvan gel, can enhance the healing process of second-degree burns. In a future study, the efficacy of the optimal dose of the carrageenan gel could be examined using an alternative gelling agent. It may also be important to explore the potential synergistic effects of the two polysaccharides, carrageenan at 10% w/w and ulvan at 5% w/w, either in combination or not, with a therapeutic drug.

## Figures and Tables

**Table 1:** The description of the different study groups (n = 7) according to the administered gels and their concentrations (w/w).

Study Groups	Treatment
Control	no treatment
Vehicle	gel only with the excipients (PIL)
Ulvan 1.5%	1.5% ulvan gel
Ulvan 5%	5% ulvan gel
Ulvan 10%	10% ulvan gel
Carrageenan 1.5%	1.5% carrageenan gel
Carrageenan 5%	5% carrageenan gel
Carrageenan 10%	10% carrageenan gel

**Table 2:** Scoring system for histopathological evaluation of burn wound healing.

	Absence	Mild	Moderate	Heavy
Inflammation	0	1	2	3
Edema	0	1	2	3
Hyperkeratosis	0	1	2	3
Wound thickness	0	1	2	3
Ulceration	0	1	2	3
Necrosis	0	1	2	3
Parakeratosis	0	1	2	3

**Table 3:** Wound reduction equations, as derived from the linear regression analysis, for the different treatments and concentrations (w/w) regarding the first half (days 1–15) and the second half (days 15–26) of the healing process.

	Day 1–Day 15	Day 15–Day 26
Control	Y = −13.44 × X + 274.8	Y = −4.574 × X + 112.1
Vehicle	Y = −12.83 × X + 286.8	Y = −5.839 × X + 140.6
Ulvan 1.5%	Y = −13.76 × X + 293.0	Y = −5.841 × X + 140.9
Ulvan 5%	Y = −13.51 × X + 281.8	Y = −4.301 × X + 101.5
Ulvan 10%	Y = −12.06 × X + 283.6	Y = −6.215 × X + 148.8
Carrageenan 1.5%	Y = −14.20 × X + 288.3	Y = −3.866 × X + 92.23
Carrageenan 5%	Y = −12.23 × X + 276.3	Y = −5.588 × X + 132.6
Carrageenan 10%	Y = −15.59 × X + 271.6	Y = −2.656 × X + 62.78

**Table 4:** Histopathological results according to different treatments: evaluation of inflammation (Inf), edema (Oed), hyperkeratosis (HPKe), wound thickness (WTh), ulceration and necrosis (Ulce&Ne), and parakeratosis (PKe).

Treatment	Inf	Oed	HPKe	WTh	Ulce&Ne	PKe	Total Score
Control	2.0	3.0	3.0	3.0	0	1.0	12.0
Vehicle	3.0	3.0	2.7	3.0	0	0.7	12.4
Ulvan 1.5%	2.0	2.3	2.3	2.3	0	0.3	9.2
Ulvan 5%	1.7	1.0	2.0	1.7	0	0	6.4
Ulvan 10%	2.7	2.0	2.0	2.0	0	0	8.7
Carrageenan 1.5%	3.0	3.0	2.0	2.0	0	0	10.0
Carrageenan 5%	3.0	3.0	3.0	3.0	0	0	12.0
Carrageenan 10%	1.3	1.0	1.0	1.3	0	0	4.6

## Data Availability

The raw data supporting the conclusions of this article will be made available by the authors on request.
